# Transmitral Gradient Assessment Using a Radial Coronary Catheter and Pressure Wire: The First Clinical Report

**DOI:** 10.1016/j.jscai.2021.100014

**Published:** 2022-01-31

**Authors:** Eric S. Rothstein, Augustin J. DeLago, Amit P. Amin, John E. Jayne, Aaron V. Kaplan

**Affiliations:** Dartmouth-Hitchcock Medical Center Heart and Vascular Center, Geisel School of Medicine, Lebanon, New Hampshire

**Keywords:** Hemodynamics, Left Atrial Pressure, Mitral Stenosis

## Abstract

• Single radial artery access for direct transmitral gradient measurement is feasible
• A radial TIG coronary catheter can be advanced retrograde into the left atrium
• A coronary pressure wire is delivered to the left atrium and left in place
• The catheter is subsequently withdrawn into the left ventricle
• This permits simultaneous left atrial and left ventricular hemodynamic assessment

## Introduction

Accurate measurement of the transmitral gradient (TMG) is central to the evaluation of mitral stenosis.^1^ Although noninvasive tools typically provide good estimates of TMG, direct simultaneous measurement of left atrial (LA) and left ventricular (LV) pressures remains the gold standard. However, accessing the LA using conventional transseptal puncture requires specialized expertise and adds procedural risk.^2^ Fa’ak et al previously described measurement of LA pressure using a coronary catheter placed retrograde across the mitral valve from the LV with counterclockwise rotation.^3^ Building on this technique, we report measurement of a TMG using a pressure wire positioned in the LA through a coronary catheter.

## Case

A 67-year-old woman presented with a 2-year history of progressive dyspnea with exertion. Echocardiographic evaluation was limited secondary to poor imaging windows, but demonstrated severe mitral annular calcification with a calculated mitral valve area of 1.5 cm^2^ (pressure half time). The patient was referred for cardiac catheterization for definitive evaluation.

**Fig. 1:**
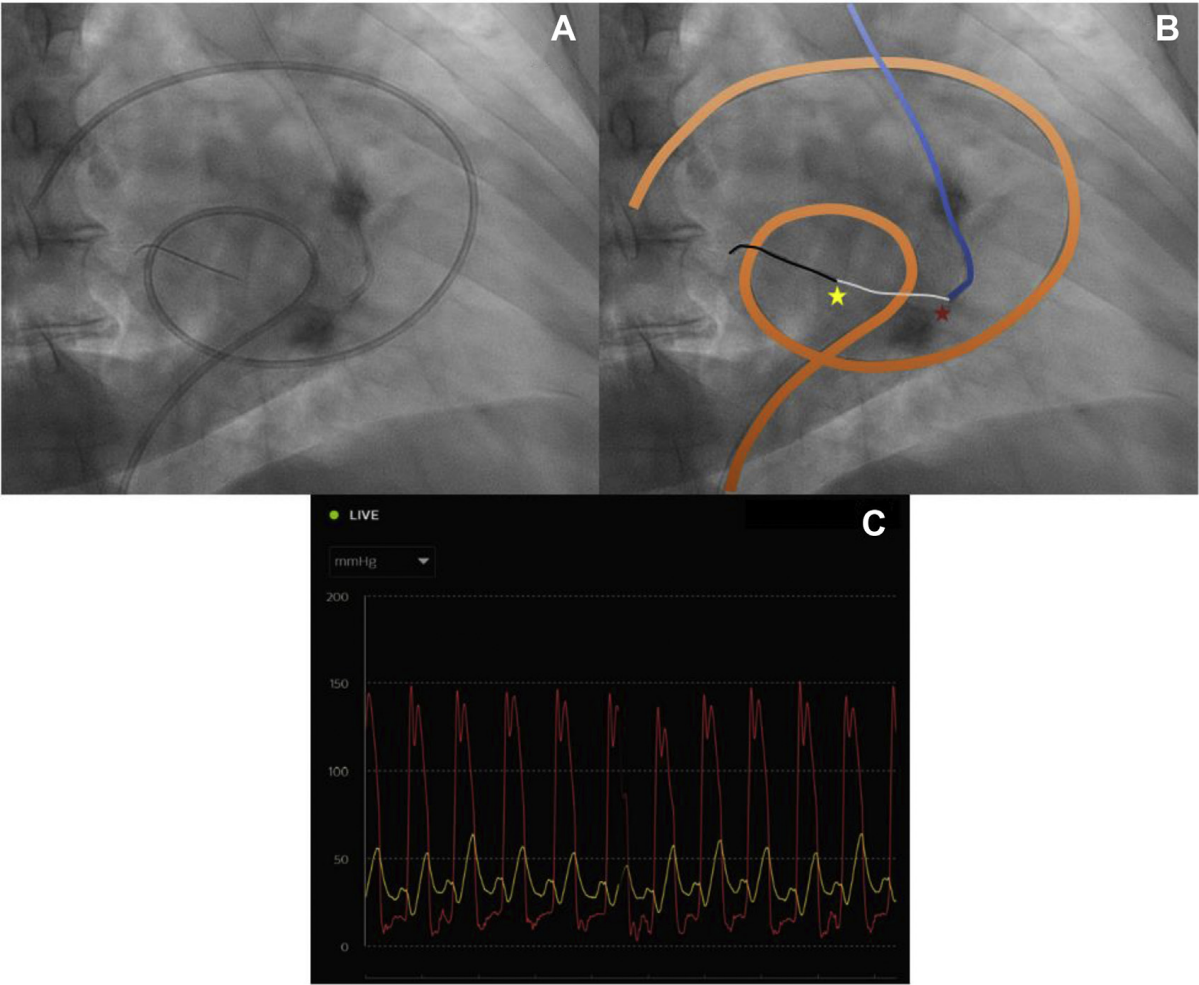
Panel A: Fluoroscopic image in the left anterior oblique projection with the pulmonary arterial catheter placed via a femoral approach, the TIG catheter placed via a radial approach into the LV, and the pressure wire in the LA. Panel B: Enhanced fluoroscopic image with the PA catheter highlighted in gray, the TIG catheter in blue, and the pressure wire in black. The yellow star indicates the pressure wire segment where LA pressure is measured; the red star indicates the distal end of the TIG catheter where LV pressure is measured. Panel C: Hemodynamic panel showing simultaneous LV (red) and LA (yellow) tracings. LA, left atrial; LV, left ventricular; TMG, transmitral gradient.

Right heart hemodynamics were obtained using a femoral approach. Selective coronary angiography was performed using a coronary guide catheter (TIG OPTITORQUE 6 Fr, Terumo Medical) via a radial approach. The guide catheter was advanced across the aortic valve in a retrograde fashion using the standard technique. In the right anterior oblique projection, the guide catheter was rotated counterclockwise and gently withdrawn until it crossed the calcified mitral annulus, which was confirmed by the presence of a LA wave form. A pressure wire (OmniWire, Philips Healthcare) was tracked through the catheter into the LA. After normalization, the guide catheter was withdrawn into the LV while maintaining the position of the distal wire segment, allowing for simultaneous LA-LV pressure measurement (Fig. 1). Hemodynamic tracings were printed and the TMG was measured using a hand planimeter. The mitral valve area was calculated to be 1.3 cm^2^ using the Gorlin equation.^4^

## Discussion

Accessing the LA using a diagnostic catheter retrograde across the mitral valve has been reported previously^3^ as has the use of a coronary catheter-pressure wire technique to measure transaortic gradients.^5^ We believe this to be the first report of TMG measurement using a pressure wire placed retrograde into the LA via a coronary catheter. Our experience confirms the feasibility of this approach, which avoids the need for transseptal puncture. Further experience will be required to characterize risks associated with this procedure as well as “tips and tricks” that facilitate retrograde crossing and the ability to obtain tracings without high-grade ectopy. A current limitation of this method is the lack of integrated software for gradient measurement and valve area calculation, leading us to use a hand planimeter. Dedicated software embedded within pressure wire platforms will facilitate adoption of this approach.

## Conclusion

Direct measurement of the TMG can be performed during cardiac catheterization using single arterial access with a pressure wire delivered in a retrograde fashion via a standard coronary catheter, obviating the need for transseptal puncture.
